# Midwives’ Attitude and Barriers of Evidence Based Practice in Maternity Care

**DOI:** 10.21315/mjms2018.25.3.12

**Published:** 2017-11-20

**Authors:** Elham Azmoude, Maryam Aradmehr, Faezeh Dehghani

**Affiliations:** 1 Department of Midwifery, Torbat Heydariyeh University of Medical Sciences, Torbat Heydariyeh, Iran; 2 Department of Midwifery, School of Nursing and Midwifery, Sabzevar University of Medical Sciences, Sabzevar, Iran; 3 Student Research Committee, Torbat Heydariyeh University of Medical Sciences, Torbat Heydariyeh, Iran

**Keywords:** evidence-based practice, attitude, barriers, midwives, maternity care

## Abstract

**Objectives:**

Midwives have a crucial role in providing optimal care for pregnant women. One of the most important policies for quality improvement in maternity care is implementation of evidence-based practice. However, the application of evidence-based practice within the maternity health care setting faces many challenges. The purpose of this study was to describe Iranian midwives’ attitude and perceived barriers of evidence based practice in maternity care.

**Methods:**

In this descriptive, cross-sectional study, a census sample of 76 midwives from two public hospitals and urban health centers in Torbat Heydariyeh, a city east of Iran were surveyed. Data collection tools were two reliable and valid questionnaires that measure midwives’ attitudes and barriers of implementation of evidence-based practice. Data were analysed using SPSS version 16.

**Results:**

The mean age and years of experience were 29.30 ± 4.86 and 5.22 ± 4.21 years, respectively. The mean score of attitude was 40.85 ± 4.84 (range = 30–60). This study also found time constraints (2.70 ± 0.92), inadequate facilities (2.64 ± 0.72), non-compilation of literature in one place (2.59 ± 0.92), lack of cooperation of physicians (2.48 ± 1.06) and the feeling of inadequate authority (2.45 ± 0.88) as the top five barriers to implementing EBP.

**Conclusion:**

Survey participants demonstrated a positive attitude toward EBP. Organisational comprehensive strategies such as time efficiency, adequate material and human resources, familiarity with organisations such as the Cochrane Collaboration and managerial support for increasing professional legitimate authority are recommended to promote the use of Evidence-Based Practice in Iran.

## Introduction

Over 1,170,000 women give birth annually in Iran (1). Improving maternal health in pregnancy and childbirth period is one of the major developmental aims of the fifth millennium of the World Health Organization (2, 3). Maternal mortality rate as one indicator of the quality of maternal health care has been considered to be a matter of public health concern worldwide, especially in developing countries (4, 5).

Based on the present evidence, the use of routine interventions without valid indications in the management of obstetric complications have contributed to the dramatic increase in this indicator (6). Therefore, World Health Organization has proposed several key strategies, instead of the use of routine interventions in maternity care, such as to apply the best evidence in clinical care (7). This approach, referred to as evidence-based practice (EBP) is defined to “identify, critically appraise and apply the best available evidence in making decisions about the care of patients” (8). This method has been recognised as the gold standard to provide high quality and safe care and optimal clinical outcomes (9).

Based on the literature, the success in adoption of EBP depends on several factors like knowledge and attitude of individuals toward EBP and their perception of existing barriers (9–12). For instance, a study done in Iran by Farokhzadian et al. (9) found that the nurses’ attitude toward EBP was unfavorable and two biggest barriers militating against the implementation of EBP were “difficulty judging the quality of research papers and reports” and “difficulty in determining the applicability of research findings.” Therefore, there is a need to study the health staff attitudes toward EBP and existing barriers in order to provide insight on ways to deal with these barriers and to develop educational programs, thereby helping to close the gap between research and practice (13, 14).

Despite the achievement of these results, most research focused on the nurse’s and physician’s view (9, 15–18). In addition, few studies have analysed other medical professionals such as midwives. This is why in Iran, prenatal care is often offered by midwife (19). Midwifery is a research-based profession (20). Moreover, in order to achieve the best maternity care goal, it is also necessary that midwives use the best available research on the safety and effectiveness of specific practices to help guide maternity care decisions and to facilitate optimal outcome in mother and newborn (21). The main purposes of the present study were to assess midwives’ attitudes toward EBP and to determine their perceptions of related barriers to the use of evidence-based maternity care. The present study is significant to improve maternity care quality and offer policy recommendation and other strategies.

## Materials and Methods

This is a cross-sectional study, which utilised a census sample of 99 employed midwives in two public hospitals and all four urban health centers in Torbat Heydariyeh, a city located in Khorasan-Razavi Province, Iran.

The study subjects included all midwives, who agreed to participate in the study, and currently have at least six months working experience. Those who were on sick or maternity leave during this period were excluded from the study. In order to collect the necessary information, an instrument was designed to investigate the participants’ sociodemographic and professional characteristics such as education level, job title, years of experience, experience of training in EBP, level of proficiency in English language, statistical analysis and use of electronic information database.

To assess attitude, an instrument was also used, that was constructed and used by Azza and Hussein (22) in a study on nursing educators in Egypt. It consists of 13 items to identify the attitude of the participants. Each item is scored on a 5-point Likert’s scale ranging between 1 (completely disagree) to 5 (completely agree). Scores for negative statements are reversed. A higher score indicates a more positive attitude toward EBP (22). This scale was translated into Farsi by two translators independently. Then back translation was performed by a translator who had not seen the original questionnaire. The content and face validity of this questionnaire were confirmed by a panel of experts in the field. Study of Azza and Hussein (22) suggests adequate internal consistency reliability of this scale (Cronbach’s alpha = 0.724). In this study, this scale had Cronbach’s α = 0.71, indicating acceptable to excellent levels of internal consistency.

Funk et al.’s (23) Barriers Scale was employed to explore the perceived barriers to research use. This scale consists of 31 items and four subscales: i) characteristics of the Adopter (midwives) (eight items), ii) characteristics of the setting (Organisation) (eight items), iii) characteristics of the esearch (Innovation) (six items), and iv) characteristics of the Presentation (communication) (six items). The items are randomly arranged throughout the questionnaire without identification of the factor titles. One item was not included in any of the four subscales.

The answers were given on a 5-point Likert Scale: 1 = no extent; 2 = to a little extent; 3 = to a moderate extent; 4 = to a great extent and 5 = no opinion. To perform the analysis, score of “no opinion” wasn’t calculated in the mean. The score of each subscale was calculated by summing the scores of different items and then dividing by the number of items in the subscale.

The face and content validity of the original scale has been established by Funk et al. (23). Findings of Funk et al.’s study also suggest moderate to good internal consistency reliability for four subscale scores [α range = 0.65–0.80] (23). Content validity and clarity of the Persian version of this scale were confirmed in some studies in Iran (24, 25). In this study, internal reliability was also established with Cronbach’s alpha coefficient of 0.69–0.74 for all subscales. Each of the participating midwives received information about the background and aim of the study, and it was emphasised that participation was voluntary and anonymous prior to participation. The questionnaires were distributed and answered in a 15-min private session at the beginning of the work shift in a private room.

## Ethical Consideration

The study was performed after approval from the Ethical Board Committee of Torbat Heydariyeh University of Medical Sciences, Torbat Heydariyeh, Iran (approval number IR.THUMS.REC.1394.2). Furthermore, participants were given information about the purpose of the study, and they were included in the study only after they have signed written informed consent form. The midwives were also assured of anonymity and confidentiality of the data and they were also reminded that they could withdraw from the study at any point.

## Data Analysis

Both descriptive and analytical statistics including Spearman correlation analysis and Man-Whitney test were performed using Statistical Package for Social Sciences (SPSS) software version 16.0. A *P*-value < 0.05 was considered statistically significant.

## Results

The total community population was 99 out of which 76 midwives participated in the study, 42 from hospitals and 34 from health centers, giving a response rate of 77.7%. The age of the participants ranged from 22 up to 43 years, with a mean of 29.30 ± 4.86 years. Most of the participants were employed with Bachelor’s degree (93.4%) as their highest educational degree and 6.6% had a master’s degree. The years of experience ranged from 6 months to 18 years (mean ± SD = 5.22 ± 4.21 years). Less than half of the respondents reported that they attend formal training on EBP (44.7%). In addition, the results showed that the mean score of proficiency in English language, statistical analysis and use of electronic information databases were 2.69 ± 0.56, 2.16 ± 0.75 and 2.69 ± 0.68 (ranged from 1–4), respectively.

The mean score of attitude was 40.85 ± 4.84. Moreover, the mean scores of all items in this scale ranged from 2.19–4.18. Midwives’ attitudes toward “the application of EBP improves patient health care outcomes,” and “EBP encourages patient-centered care” had the highest mean scores (4.18 ± 0.53 and 4.02 ± 0.63), whereas “EBP is a waste of time” and “EBP is too tedious and impractical” resulted in a lower mean attitude score in comparison to the other items (Table 1). Based on the results of this study, there were also no significant differences in midwives’ reported attitude scores across level of education (*P* = 0.186), job place (*P* = 0.066) and experience of training in EBP (*P* = 0.335) based on the results of Kruskal–Wallis and Mann–Whitney U-tests. In addition, Spearman correlation analysis showed that age and years of experience don’t have any significant correlation with mean score of attitude. The relationship between the participants’ level of proficiency in English language, statistical analysis and use of electronic information database with attitude score were also not significant (Table 2).

The results in Table 3 indicated that six out of the top 10 barriers were related to organisational barriers, two to qualities of research, one to communication of research and one to adopter characteristic.

Additionally, as shown, the three greatest barriers to research utilisation are “The nurse does not have time to read research” (mean = 2.70), “The facilities are inadequate for implementation” (mean = 2.64) and “The relevant literature is not compiled in one place” (mean = 2.59).

Overall, the most prominent barriers were the organisation subscales (mean ± SD = 2.51 ± 0.54) and the innovation (mean ± SD = 2.33 ± 0.56). The barriers concerning communication (mean ± SD = 2.28 ± 0.62) and adopter (mean ± SD = 2.22 ± 0.56) were in the next rank.

## Discussion

This was the first available study that aimed to evaluate attitudes and perceived barriers toward evidence-based maternity care in Iranian midwives. The present study indicates that midwives had positive attitudes toward EBP. This finding is supported by the findings of Mehrdad et al. in Iranian nurse (26). Furthermore, this result also supports the finding of other studies which showed that most of the health care professionals in other countries had a positive attitude toward EBP (16, 18, 27, 28). On the other hand, the attitudes of health care professionals toward EBP were negative in a few studies (9, 29, 30). Consequently, the desirable attitude as one of the fundamental prerequisite of implementing EBP is located in a favorable position in this population.

Attitudes toward EBP were not different according to individual and professional characteristics. For instance, the finding showed that there was no difference between MSc and BSc degree midwives regarding attitudes toward EBP. This is in line with the finding of the study conducted by Majid et al. (16). On the contrary, Eid AbuRuz et al. found that Jordanian nurses with MSc degree have a more positive attitude (31). In Iran, medical students learn specialised courses about research at master level. However, the reason for this contradiction may be attributed to the low number of midwives with master degree in this study. In addition, MSc and BSc degree midwives are working in the same place, which may have effect on their attitude. Previous participation in EBP training was not associated with positive attitude toward EBP. Moreover, the correlation between mean scores of attitude toward EBP with proficiency in English language, statistical analysis and use of electronic information databases were not significant (*P* < 0.05). These professional variables are probably more influenced by midwives’ knowledge than their attitudes.

This study also allows us to identify perceived barriers of implementing EBP among midwives. The five top ranked barriers included limitations of time, inadequate facilities, non-compilation of literature in one place, lack of cooperation of physicians and the feeling of inadequate authority. Similarly, the major barriers to research utilisation in another study among Iranian nurses were that the nurses do not have time to read research, facilities are inadequate for implementation and nurses do not feel they have enough authority to change patient care procedures (25). The results of a review from 10 studies about research utilisation in Iran also showed that “Time limitation” and “insufficient facilities” were the main barriers (13). Several other studies also highlighted the lack of time as the greatest barrier to adopting EBP in the other medical groups and in different parts of the world (32–34). Similar to other countries, the midwifery shortage and heavy workload of midwives are common issues in Iranian hospitals and health system (35). Therefore, providing adequate human resources for maternity care delivery would be fundamental.

The second barrier identified in this study was “The facilities are inadequate for implementation.” Adequate facilities, including material resources (such as access to computer, electronic databases and journal/ libraries) or human resources (such as access to clinical specialists) are crucial to the successful implementation of EBP. The lack of compilation of literature in one place was determined as the third barrier. This could be attributed to low level knowledge of research and extracting information from literatures. Designing strategies that promote midwife’s knowledge about extracting data, compiling recent evidence in accessible places, access to facilities such as internet, familiarity of midwives with organisations such as the Cochrane Collaboration and the National Institute for Health and Clinical Excellence that provide access to organised and synthesised research evidence, may serve as facilitator in research utilisation (36).

The two next barriers were related to the belief that “Physicians will not cooperate with implementation” and “The midwives does not feel they have enough authority to change patient care procedures.” Obstetricians and midwives, as members of health team have complementary roles in providing antenatal care. The effectiveness of this team depends on the extent to which they held similar perspectives on how to provide care (37). In Iran, midwives have little authority for providing maternity care. Many of their health services are not covered by insurance. So they even have restrictions to offer an ultrasound screening during the care of a pregnant woman. Therefore, midwives require managerial support to increase their professional legitimate authority in clinical decision-making. These two items were ranked in the list of the first three barriers in some studies (25, 38–41). On the other hand, the Chinese nurses in Chien et al’s study did not perceive that ‘physicians will not cooperate with implementation’ which was an important barrier to research utilisation (42). Overall, organisation characteristic is ranked as the top barrier in all. This finding is consistent with many earlier studies, where the greatest barriers were about the organisational characteristic (15, 42, 43). In order to improve the quality of care offered to pregnant and childbearing women, organisational support should be considered as a significant factor in enhancing EBP implementation.

This study had some limitations. First, the use of self-reported questionnaire for data gathering which can result in misclassification bias. In addition, it is necessary to note that the cross-sectional design cannot provide strong evidence about any perceived barrier. Finally, small sample size may have limited the generalisability of the findings.

## Conclusions

Based on the findings of the current study, Iranian midwives show a positive attitude toward implementing EBP. Moreover, the findings of this study indicate that limitations of time, inadequate facilities, non-compilation of literature in one place, lack of cooperation of physicians, the feeling of inadequate authority were top barriers of implementing evidence-based maternity care. To enhance the implementation of EBP, strategies should be placed to minimise barriers such as providing adequate material and human resources, familiarity with organisations such as the Cochrane Collaboration and managerial support for increasing professional legitimate authority.

## Figures and Tables

**Table 1:** Midwives’ attitudes toward evidence based-practice

Item no	Attitude Scale Items	Mean ± SD
1	Current research findings are useful in the provision of day to day nursing practice	3.53 ± 0.77
2	The adoption of EBP places too many demands on my workload	3.10 ± 1.10
3	The application of EBP improves patient’s healthcare outcomes	4.18 ± 0.53
4	EBP encourages patient-centered care	4.02 ± 0.63
5	I dislike having my clinical/academic practice questioned.	3.32 ± 1.12
6	EBP is a waste of time	2.19 ± 0.92
7	I stick to the traditional methods rather than changing to new methods of research in patient care	2.60 ± 0.96
8	It is not easy to relate research findings to academic practice	3.13 ± 0.92
9	The importance of EBP is exaggerated	3.06 ± 0.85
10	EBP is too tedious and impractical	2.55 ± 0.98
11	EBP is not feasible in this organisation	2.90 ± 0.89
12	Human views and experiences are more valued than evidences from research	3.14 ± 1.06
13	The clinical environments do not stimulate the application of EBP	3.06 ± 0.89

**Table 2:** Correlations between demographic and professional characteristics and evidence-based attitude

Variable	Attitude score	

	*r*	*P*
Age	−0.036	0.757
years of experience	0.102	0.384
level of proficiency in English language	0.190	0.100
level of proficiency in statistical analysis	0.217	0.062
level of proficiency in using of electronic information database	0.151	0.206

**Table 3:** Barriers of implementing EBP

Rank	Factor	Barrier Item	Mean	SD
1	O	The midwives does not have time to read research	2.70	0.92
2	O	The facilities are inadequate for implementation	2.64	0.72
3	C	The relevant literature is not compiled in one place	2.59	0.92
4	O	Physicians will not cooperate with implementation	2.48	1.06
5	O	The midwives does not feel she has enough authority to change patient care procedures	2.45	0.88
6	I	The conclusions drawn from the research are not justified	2.45	0.97
7	O	Other staff are not supportive of implementation	2.44	.96
8	O	Administration will not allow implementation	2.43	1.04
9	I	The literature reports conflicting results	2.42	1.01
10	A	The midwives is unwilling to change/try new ideas	2.42	1.01
11	A	The midwives is unaware of the research	2.41	0.72
12	A	There is not a documented need to change practice	2.40	0.96
13	O	There is insufficient time on the job to implement new ideas	2.40	0.99
14	I	The research has not been replicated	2.40	0.80
15	I	Research reports/articles are not published fast enough	2.39	0.85
16	A	The midwives does not see the value of research for practice	2.34	1.08
17	C	Research reports/articles are not readily available	2.31	0.92
18	C	Statistical analyses are not understandable	2.31	0.92
19	C	Implications for practice are not made clear	2.31	0.81
20	C	The research is not reported clearly and readably	2.30	0.99
21	A	The midwives sees little benefit for self	2.28	0.96
22	O	The midwives feels results are not generalisable to own setting	2.25	0.93
23	I=NO	The amount of research information is overwhelming	2.24	0.91
24	I	The midwives is uncertain whether to believe the results of the research	2.21	1.00
25	I	The research has methodological inadequacies	2.18	0.88
26	A	The midwives does not feel capable of evaluating the quality of the research	2.18	0.89
27	A	The midwives feels the benefits of changing practice will be minimal	2.16	0.85
28	C	The research is not relevant to the nurse’s practice	2.10	0.92
29	A	The midwives is isolated from knowledgeable colleagues with whom to discuss the research	2.04	0.87
